# Incidental finding of diffuse cavernous rectal haemangiomatosis during bowel cancer screening

**DOI:** 10.1186/s12876-019-1118-6

**Published:** 2019-11-27

**Authors:** Kushala W. M. Abeysekera, Daniel S. Pearl, Paul Burn, Andrew Lowe

**Affiliations:** 1 Gastroenterology Department, Musgrove Park Hospital, Parkfield Drive, Taunton, Somerset TA1 5DA UK; 2 Gastrointestinal Radiology Department, Musgrove Park Hospital, Taunton, UK

**Keywords:** Case report, Diffuse cavernous haemangiomatosis of the rectum, Endoscopy, Magnetic resonance imaging

## Abstract

**Background:**

This case seeks to highlight to endoscopists a rare benign disorder that may be encountered during endoscopy. Clinicians may be tempted to biopsy, which could lead to a catastrophic gastrointestinal haemorrhage.

**Case presentation:**

A 66-year-old asymptomatic Caucasian male was referred for colonoscopy with a positive faecal occult blood test as part of the UK national bowel cancer screening programme. Relevant past medical history included atrial fibrillation for which he took Dabigatran. He had a normal haemoglobin, mean cell volume, platelet and clotting function.

During colonoscopy, an unusual vascular pattern encompassing the entire rectum extending to the rectosigmoid junction was noted at intubation. The lesion demonstrated confluent circumferential purple discolouration indicating venous blood supply, with heaping up of the mucosa involving the entire rectum and rectosigmoid junction. There was no corresponding history of venothromboembolic disease or liver disease. The patient proceeded to have computed tomography (CT) which revealed a considerably thickened rectosigmoid wall with multiple small rounded punctate calcifications within it, and no other visceral involvement. Subsequent magnetic resonance (MR) scan of the pelvis demonstrated extensive diffuse thickening of the rectum and lower sigmoid with intermediate to high T2 signal, and an internal architecture of multiple ‘grapelike’ lobulations.

**Conclusion:**

The findings were consistent with diffuse cavernous haemangiomatosis of the rectum (DCHR), an extremely rare benign submucosal vascular intestinal tumour originating from the dentate line. Misdiagnosis of DCHR is common and the macroscopic appearance of DCHR can mimic varices, haemorrhoids, polyps or proctitis. MR imaging is the gold standard for diagnosis. Common presentation is with haematochezia due to mucosal wall erosion. The treatment of choice for symptomatic DCHR is pull-through transection and colo-anal anastomosis. This case seeks to highlight a rare disorder that can be encountered incidentally during lower GI endoscopy. Injudicious biopsy is potentially catastrophic. In a patient who endoscopically has evidence of a DCHR, we advocate MR pelvis assessment to clarify the nature of the lesion to guide future management if required. The patient discussed remains well, asymptomatic, with no evidence of iron deficiency anaemia.

## Background

This is a cautionary case for gastroenterologists who may encounter this rare benign vascular malformation during endoscopy and be tempted to biopsy, which could have severe consequences. The case provides an alternative differential to rectal varices, in the absence of portal hypertension.

It also raises awareness of a condition that is associated with a significant lag between symptoms to diagnosis, despite diagnosis being straightforward with magnetic resonance imaging assessment.

## Case presentation

A 66-year-old Caucasian male was referred for colonoscopy having had a positive faecal occult blood test (FOBT) as part of the national bowel cancer screening programme performed in the UK. At screening the patient was asymptomatic and denied rectal bleeding. He was not anaemic.

The endoscopist noted a past medical history of ischaemic heart disease, systemic lupus erythematosus, atrial fibrillation and hypercholesterolaemia, for which his medication included Dabigatran. The patient was a retired labourer, normally fit and well with a performance status of 1.

### Investigations

**Fig. 1:**
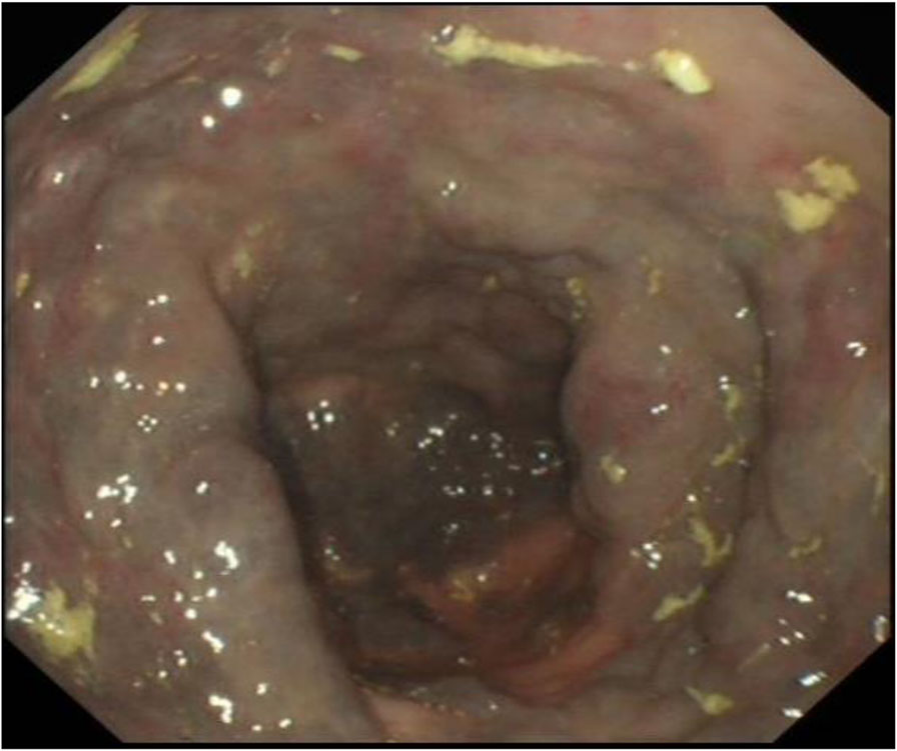
Endoscopic findings of rectum with vascular congestion and diffuse circumferential grape like lesion. Circumferential involvement of the rectal wall with heaped up and engorged folds with a purple discolouration

**Fig. 2:**
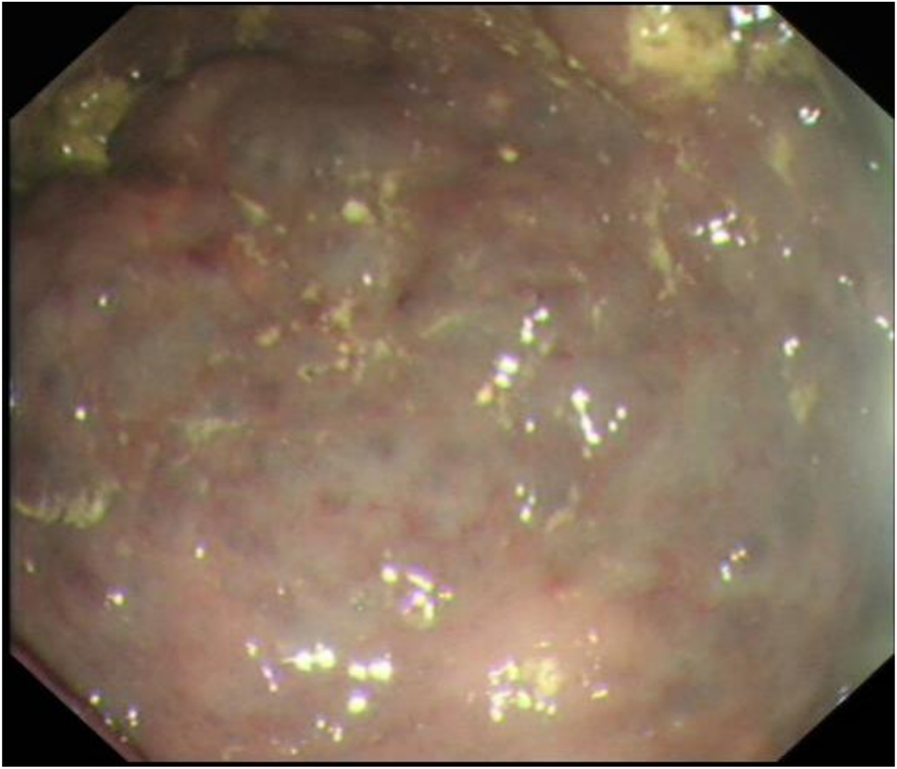
Endoscopic findings of rectum. Nodular areas of purple coloured rectal wall

**Fig. 3:**
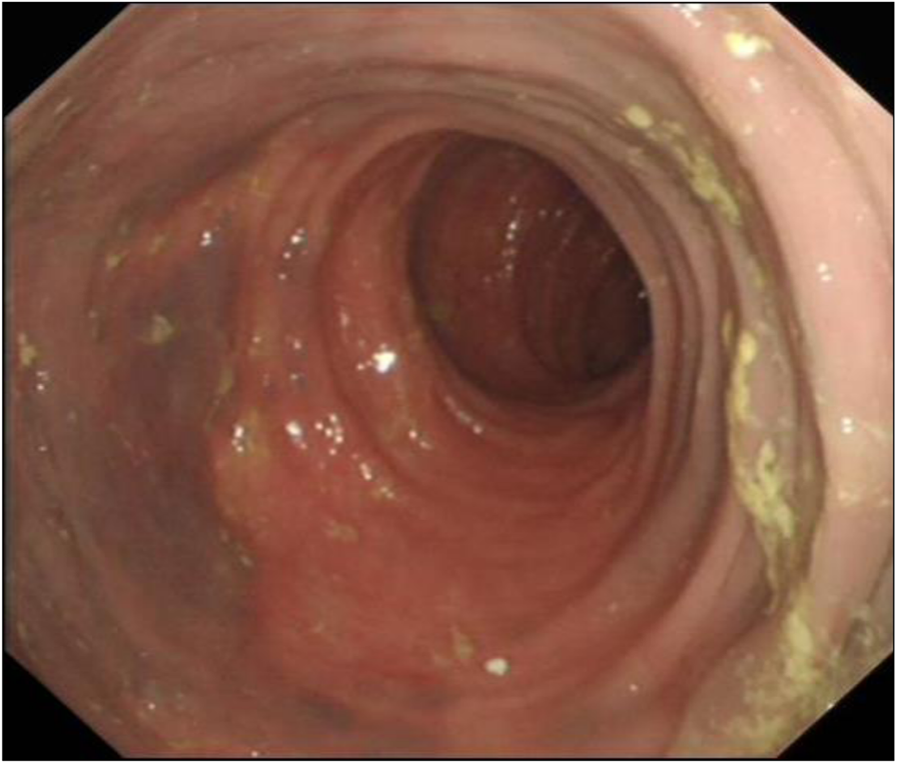
Endoscopic image of rectosigmoid junction. Well demarcated involvement of the rectal wall characterised by a purple discolouration. The mucosa was intact with no evidence of ulceration or active bleeding

Colonoscopy was carried out using Olympus standard colonoscope with CO_2_ insufflation and pump irrigation containing simethicone, under Entonox gas. At intubation an unusual vascular pattern encompassing the entire rectum up to the rectosigmoid junction was noted. At this point a decision was taken with the patient to proceed with full colonoscopy. The lesion was characterised by confluent circumferential purple discolouration indicating venous blood supply, with heaping up of the mucosa involving the entire rectum (see Figs. 1 & 2) and rectosigmoid junction. There was a well demarcated border with normal mucosa in the distal sigmoid colon (see Fig. 3). No mucosal ulceration or active bleeding was seen.

Macroscopically, the pattern within the rectum resembled extensive rectal varices, although the patient did not have liver disease or venothromboembolic disease and was not known to have any cutaneous or visceral vascular malformation.

**Fig. 4:**
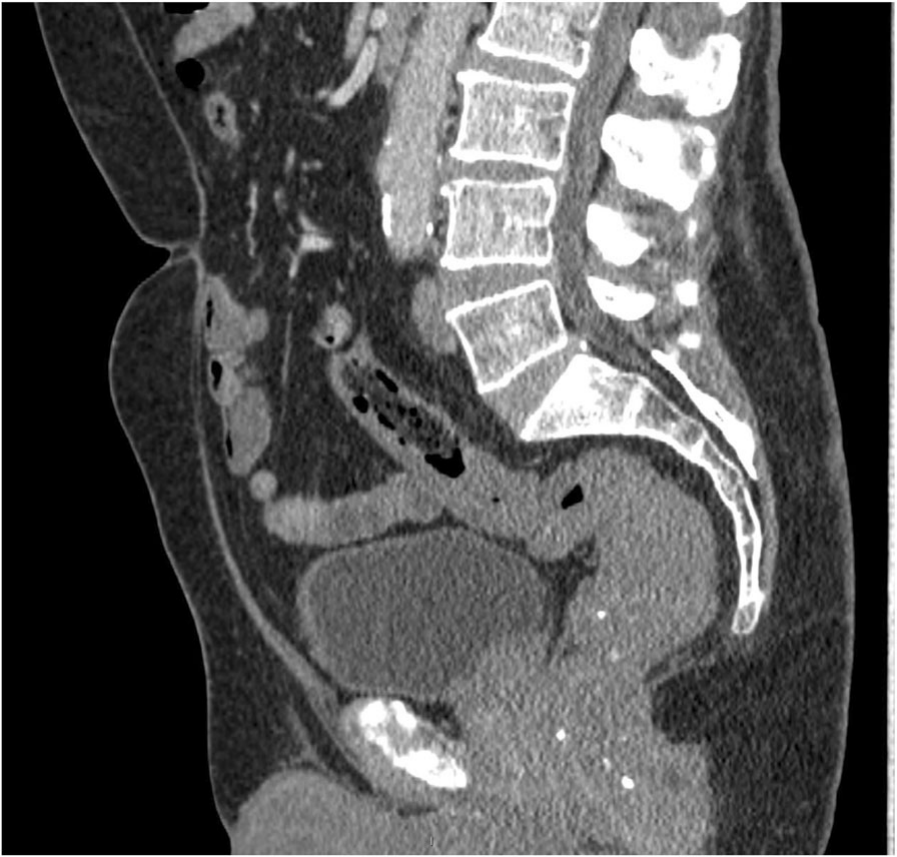
Sagittal contrast-enhanced CT abdomen and pelvis. Irregular and eccentric thickening of the rectum. Multiple calcified phleboliths. Relatively abrupt transition to normal rectal wall at the rectosigmoid junction

The patient proceeded to axial imaging with computed tomography (CT) to further evaluate this abnormality. The CT demonstrated a considerably thickened rectosigmoid wall with multiple small rounded punctate calcifications seen within it (see Fig. 4). There was no evidence of other visceral involvement or cutaneous lesions.

**Fig. 5:**
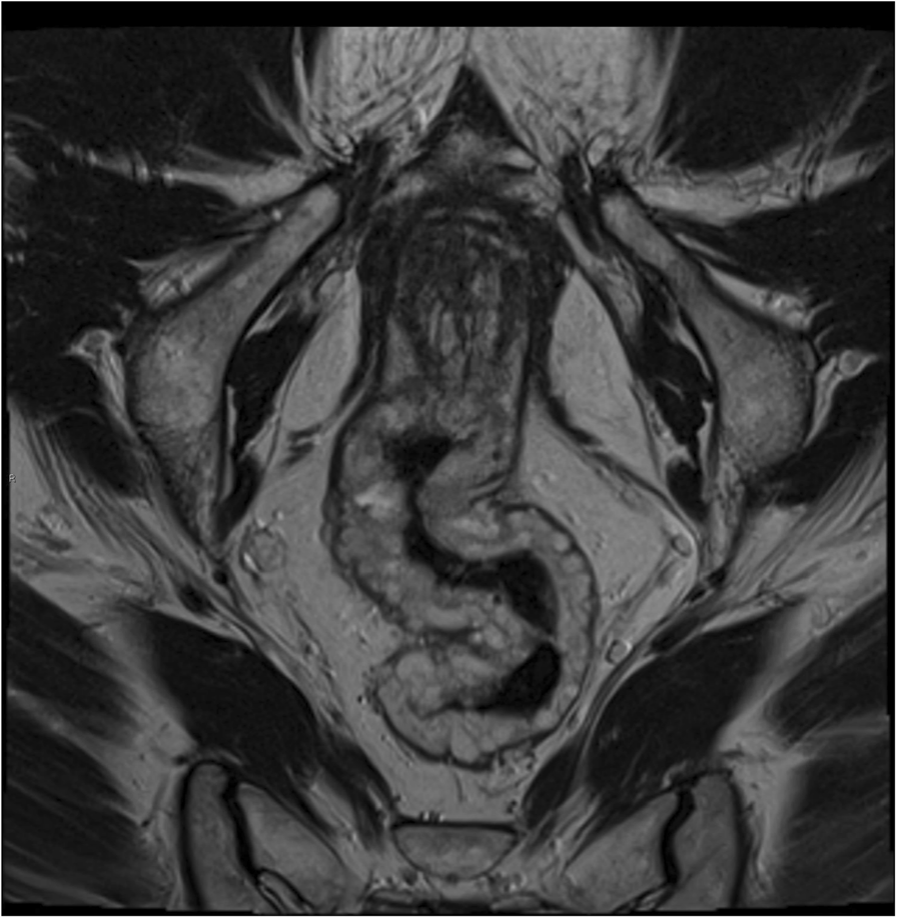
Coronal T2 weighted MRI pelvis. Nodular, predominantly high signal thickening of rectal wall

**Fig. 6:**
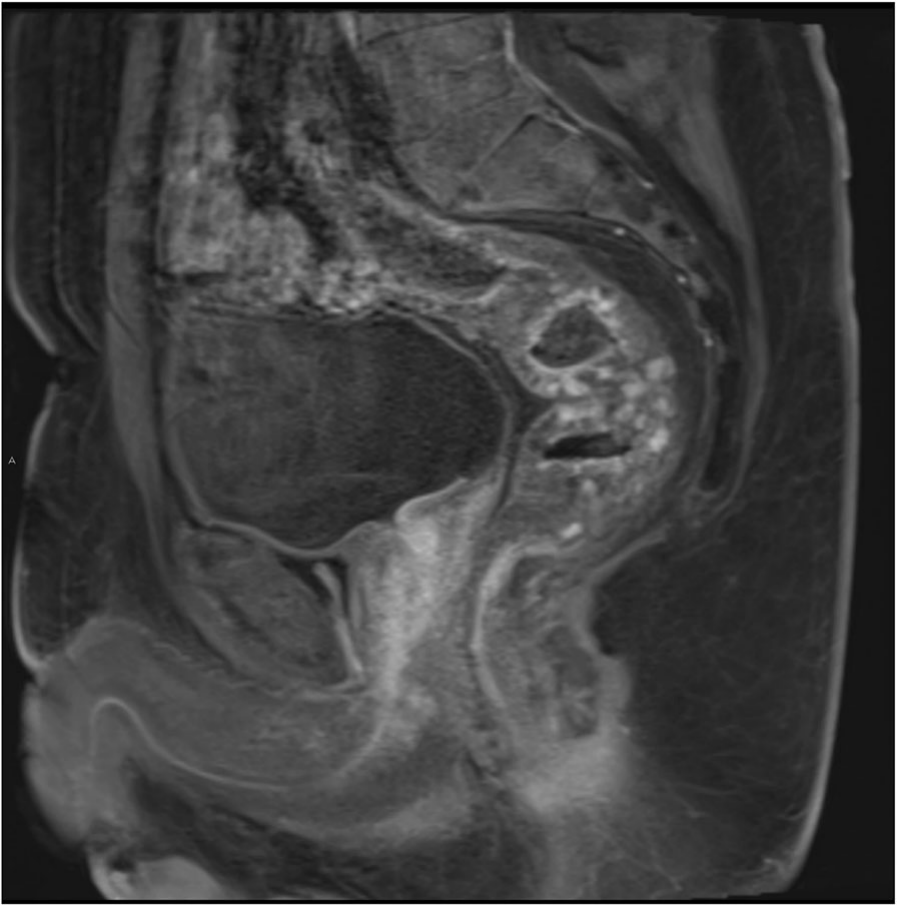
Sagittal T1 weighted MRI pelvis (early phase post-contrast fat saturated). Intense nodular enhancement of the rectal wall

To further characterise the CT findings a magnetic resonance (MR) scan of the pelvis was performed which demonstrated extensive diffuse thickening of the rectum and lower sigmoid with intermediate to high T2 signal, and an internal architecture of multiple ‘grapelike’ lobulations. The outer margin was well-defined, largely with a low signal wall indicating that the pathology was confined by the muscularis propria. Incidentally, the abnormality extended inferiorly, into the right side of the anal canal posteriorly, between the internal and external sphincter. Following enhancement, there was initially some nodular mucosal enhancement followed by progressive enhancement of the multiple discrete lobulations with infilling (see Figs. 5 and 6). Incidentally there was also a patchy intermediate signal in the prostate with avid peripheral zone enhancement. His prostate specific antigen was normal.

## Discussion and conclusions

The appearance of the CT and MR findings combined with the macroscopic appearance on colonoscopy suggested a morphology highly consistent with diffuse cavernous haemangioma of the rectum (DCHR).

DCHR is a rare benign vascular tumour of the large intestine, of unknown prevalence. First described in 1839, approximately 350 cases have been reported in literature to date [1, 2]. Colonic haemangiomas are associated with a variety of conditions including Blue rubber bleb nevus syndrome, Klippel-Trenaunay syndrome and Osler-Weber-Rendu syndrome. These syndromes are associated with haemangiomas throughout the alimentary canal and skin [3]. Conversely, DCHR is not associated with cutaneous haemangiomas and typically originates from the dentate line; distribution is confined to the rectosigmoid and rectal mesentery [4].

Eighty percent of all colonic haemangiomas are cavernous, the other major type being capillary [5]. Histologically these lesions arise from the submucosa, containing dilated tortuous blood vessels with a propensity for forming vascular sinuses [6]. DCHR lesions have a thin wall containing either a single or multiple flattened layers of endothelial cells with no capsule [7]. Phleboliths form within the haemangiomas secondary to blood flow stasis/thrombosis and subsequent chronic inflammation [8].

DCHR appears to have an equal sex distribution, and symptomatic patients tend to present in youth [7]. The vast majority of patients with DCHR are asymptomatic, as with our patient. Haematochezia can occur secondary to erosion of the haemangioma through the mucosa into the gastrointestinal lumen. This was likely the cause of his positive FOBT. Chronic iron deficiency anaemia, bowel obstruction, intussusception, perforation, and consumptive coagulopathy mimicking disseminated intravascular coagulation have all been reported as complications of DCHR in literature [4, 6, 9, 10].

Misdiagnosis of DCHR is common, with one Shanghai case series of 17 patients finding a mean delay time between onset of symptoms and diagnosis of 17.63 years [4]. To compound matters, on endoscopic evaluation, the macroscopic appearance of DCHR can mimic varices, haemorrhoids, polyps or proctitis. The latter two conditions are particularly pertinent as the inexperienced endoscopist may be tempted to obtain biopsy without considering the significant risk of haemorrhage with biopsy [5].

Evaluation of DCHR is best sought radiologically. Historically, barium enemas have played a role in identifying the length of the involved segment of colonic haemangioma. CT can depict the thickening of the rectosigmoid colon and pelvic phleboliths within the vascular sinuses of haemangiomas.

MR imaging is widely regarded as the gold standard for radiological assessment of DCHR, in view of its superior soft tissue resolution and multi-planar facilities [11]. The rectosigmoid wall in DCHR demonstrates high signal intensity on T2 weighted imaging with multiple peri-rectal serpiginous structures evident [11]. The multi-planar capability allows exact dimensions to be calculated with an assessment of sphincter involvement to guide future therapy.

Multiple therapies have been trialed with sclerotherapy, endoscopic mucosal resection and radiotherapy all failing to demonstrate long term therapeutic benefit. The treatment of choice for symptomatic DCHR is pull-through transection and colo-anal anastomosis, first proposed in 1976, and now successfully performed laparoscopically [4, 12, 13]. This has the benefit of resecting the DCHR without permanent stoma formation, as would be the case with abdominoperineal resection.

This case seeks to highlight a rare disorder that can be encountered incidentally during lower GI endoscopy. Injudicious biopsy by the inexperienced practitioner, is potentially catastrophic. In addition, in view of his potential prostatic pathology, transrectal ultrasound guided biopsy of the pelvis was contraindicated. In a patient who endoscopically has evidence of a DCHR, we advocate MR pelvis assessment to clarify the nature of the lesion to guide future management if required, and to inform the patient (and by extension the clinical team) in the event of presentation to hospital with haematochezia. The patient discussed in this case reports remains well, asymptomatic, with no evidence of iron deficiency anaemia.

## Learning points

- DCHR is a rare but significant cause of rectal bleeding.
- DCHR should remain in an endoscopist’s differential as patients can wait many years to be diagnosed.
- If you suspect DCHR endoscopically, do not biopsy.
- MR imaging is the gold standard for radiological assessment of DCHR.

## Data Availability

Data sharing is not applicable to this article as no datasets were generated or analysed during the current study.

## Abbreviations

CT: Computed tomography
DCHR: Diffuse cavernous haemangiomatosis of the rectum
FOBT: Faecal occult blood test
MR: Magnetic resonance
